# Screening, Identification, and Optimization of Protease Producing *Bacillus pumilus* Strain DT-15 From Tannery Waste Disposal Site in Addis Ababa, Ethiopia

**DOI:** 10.1155/ijm/7176092

**Published:** 2025-04-29

**Authors:** Bontu Habtamu Feyissa

**Affiliations:** College of Natural and Computational Science, Department of Biotechnology, Wolkite University, Wolkite, Ethiopia

**Keywords:** bacterial isolate, optimization, protease, skim milk, tannery waste

## Abstract

Bacterial proteases are valuable enzymes that accelerate the hydrolysis of peptide bonds within protein molecules. This study aimed to screen, identify, and optimize bacteria-producing protease from a tannery waste disposal site. Then, 36 morphologically distinct bacterial isolates were obtained from the Dire Tannery waste disposal site in Addis Ababa, Ethiopia. Among these isolates, DT-15 demonstrated the highest protease activity, with a clear zone of 19.00 ± 0.75 mm on skim milk agar, indicating its efficacy as a protease producer. Further morphological and molecular characterization of the most promising isolate was conducted. Based on its 16S rRNA sequence, the most effective isolate was identified as *Bacillus pumilus*. To enhance protease production, optimization experiments were carried out, resulting in an optimal enzyme activity of 506 ± 0.037 U/mL achieved after 60 h of incubation at 37°C and pH 7, using peptone and glucose as the nitrogen and carbon sources, respectively. Thus, the isolated bacterium has the potential to be utilized for various biotechnological applications, such as leather processing, detergent formulation, and food production. Further studies could focus on its applications in industrial processes.

## 1. Introduction

Proteases are enzymes that catalyze the hydrolysis of peptide bonds within protein molecules, leading to the formation of polypeptides. These enzymes hold significant importance across various industrial sectors. Proteases account for approximately 50%–65% of the global enzyme market [1]. Their broad range of applications spans industries such as pharmaceuticals, leather processing, detergents, dairy products, brewing, food production, and waste processing [2, 3].

Proteases are found in a wide variety of plants, animals, and microorganisms [4]. However, microorganisms are particularly favored as a source for protease production due to their rapid growth, minimal space requirements, extensive biochemical diversity, genetic manipulability for producing novel enzymes, and possession of desirable characteristics for biotechnological applications [5, 6].

Microbial protease production is highly influenced by various factors, including the type of strain, nutritional sources, and physicochemical parameters such as pH, temperature, and carbon and nitrogen sources [6]. Therefore, the objective of this research is to screen and identify protease-producing bacteria, investigate the factors influencing protease production, and partially purify the protease from a soil sample obtained from a tannery waste disposal site in Addis Ababa, Ethiopia.

By focusing on tannery waste soil samples, this study aims to explore a unique ecological niche that may harbor protease-producing bacteria adapted to thrive in the presence of complex proteinaceous substrates. Screening and characterizing these bacteria will provide valuable insights into the biodiversity of protease producers in such environments. Additionally, investigating the factors affecting protease production will help optimize conditions for maximum enzyme yield, leading to enhanced industrial applications. This research aims to contribute to the field of enzyme biotechnology by uncovering new sources of protease-producing bacteria, elucidating the factors influencing enzyme production, and providing insights into the molecular identification and optimization of the isolates. The findings of this study will not only expand our knowledge of microbial proteases but also have practical implications for the development of efficient and sustainable protease production processes for various industrial applications.

## 2. Materials and Method

### 2.1. Description of the Sampling Area

Samples were collected from Dire Tannery located in the Northwestern part of Addis Ababa, Ethiopia. Dire Tannery is located at longitude and latitude of 9°03⁣′45.5⁣^″^N and 38°42⁣′44.8⁣^″^E, respectively. Dire Tannery, a part of Dire Industries, processes 600 cattle hides and 5000 sheepskins per day, with weekly outputs of around 80,000 square feet of bovine leather used for shoe uppers and 150,000 square feet of sheepskins used for shoe uppers and lining gloves and garment leather.

### 2.2. Source of Sample Collection

Soil samples were aseptically collected from the waste disposal site of Dire Tannery in Addis Ababa, Ethiopia, using a sterile spatula at a depth of 4–5 cm. The collected samples were then transferred to sterile polyethylene bags and transported to the Microbiology Laboratory at Addis Ababa Science and Technology University, Ethiopia. The samples were stored at 4°C until further processing.

### 2.3. Isolation of Bacteria

To isolate bacteria, 1 g of the soil sample was weighed, and serial dilutions were prepared in distilled water. Samples from the final two dilutions were spread-plated in duplicate on nutrient agar plates. The plates were then incubated at 30°C for 24 h. Bacterial colonies displaying unique characteristics were transferred to nutrient agar slants and stored at 4°C for preservation [7].

### 2.4. Primary Screening of Protease-Producing Bacteria

For the screening of protease producers, isolated bacterial colonies were spot inoculated on skim milk agar (SMA) medium. Subsequently, the plates underwent a 24-h incubation period at 30°C. The measurement of clearance zones around the bacterial colonies, indicative of proteolysis, was conducted using a ruler. Bacterial isolates manifesting substantial clear zones were chosen for the secondary screening.

### 2.5. Secondary Screening of Potential Protease-Producing Bacteria

Were subjected to secondary screening. Then, 1 mL of each bacterial inoculum was added to 50 mL of nutrient broth medium and incubated at 30°C under shaking conditions at 150 rpm. After 24 h of incubation, the bacterial cultures were harvested by centrifugation at 10,000 rpm for 15 min at 4°C, and the culture supernatant was collected. SMA plates (containing 2% w/v skim milk) were prepared, and wells were created using a 5-mm sterile cork borer. Then, 10 *μ*L of the culture supernatant from each isolate was loaded into the wells, and the plates were incubated at 30°C for 24 h. After incubation, the isolates capable of producing clear zones were selected based on the diameter of the clear zones.

### 2.6. Morphological Characterization of the Best Isolate

The best bacterial isolate obtained from the primary and secondary screening methods was further characterized and identified based on morphological identification. Morphological studies were carried out using Gram staining [8], motility test [9], and endospore staining [10], in addition to cultural characterization of the isolates on agar plates (which included observation for colony morphology as colony shape, size, margin, elevation, opacity, texture, and pigmentation).

### 2.7. Molecular Identification of Best Isolate

For molecular identification, genomic DNA extraction was carried out utilizing the DNA extraction method described by Wright et al. [11]. Subsequent to DNA extraction, sequence analysis was performed utilizing both the forward primer (E9F) and reverse primer (U1510R). The generated sequences undergo sequence match analysis in FASTA format through the Basic Local Alignment Search Tool (BLAST) on NCBI. The resultant FASTA file containing the sequences was then uploaded. Consensus sequences demonstrating a noteworthy match with the extensively identified data on NCBI were subsequently submitted to NCBI for the assignment of accession numbers. Phylogenetic analysis and tree reconstruction were performed using Mega 11 software. The 16S ribosomal DNA sequences of the isolate were used for the phylogenetic analysis as they are evolutionary conserved and the results were used for tree reconstruction.

### 2.8. Optimization of Conditions for Protease Production

For optimization of protease production, the growth medium contained: galactose 1% w/v, tryptone 1% w/v, CaCl_2_ 0.5% w/v, Mg 0.02% w/v, and casein 1% w/v.

For pH, the growth medium, comprising 1% casein as a substrate, undergo pH adjustments to various values using the following buffer system: sodium acetate, sodium phosphate, phosphate buffer, sodium carbonate, and glycine NaOH with pH values ranging from 4.0 to 10.0. Following the pH adjustments, the medium was subjected to incubation at 30°C for a duration of 48 h. Subsequently, cell-free culture filtrates were collected and utilized to determine protease activities.

For temperature, the production medium, containing 1% casein as a substrate and adjusted to the optimal pH, undergo inoculation with the overnight-grown selected bacterial strain. The medium was then subjected to incubation at various temperatures (25°C, 30°C, 35°C, 37°C, 40°C, and 45°) for a duration of 48 h. Following incubation, the cell-free culture filtrate was harvested from each temperature setting and employed for the evaluation of protease activity.

For carbon source, the effect of carbon source on protease production was investigated by substituting galactose in the production medium with different carbon sources, including glucose, sucrose, starch, lactose, and fructose.

For nitrogen source, the effect of various nitrogen sources, such as peptone, yeast extract, beef extract, tryptone, and urea, on protease production was investigated by substituting 1% tryptone in the production medium.

In the incubation period, to ascertain the optimal duration for achieving optimum protease production, the grown isolate was introduced into the protease production medium, which included 1% casein as a substrate. Subsequently, the medium was incubated at the optimum temperature and pH range for a period spanning 12–84 h. Samples were systematically collected at 12-h intervals to assess and identify the time point corresponding to the highest recorded protease activity.

### 2.9. Protease Assay

For protease assay, 0.5 mL of enzyme extract was mixed with 2.5 mL of 1% casein solution (prepared in phosphate buffer, pH 7.3) and incubated for 10 min at 30°C. The reaction was terminated by adding 2.5 mL of trichloroacetic acid (TCA) to the solution. The mixture was vortexed to ensure complete mixing, incubated for an additional 15 min at room temperature, and then centrifuged at 10,000 rpm for 15 min at 4°C. After centrifugation, 0.5 mL of Folin reagent was immediately added to the supernatant, vortexed, and left for 30 min at room temperature. The mixture was then used to estimate the amount of free tyrosine released, following the Lowry et al. [12] method, using tyrosine as a standard. The amount of tyrosine in the solution was measured by reading the absorbance at 660 nm. One unit of protease activity was defined as the amount of enzyme required to liberate 1 *μ*g of tyrosine per minute at 30°C. A tyrosine standard curve was prepared by dissolving different amounts of tyrosine in TCA solution [13].

 $$\begin{array}{c} \text{Enzyme activity}=\frac{\text{u mol tyrosine equivalent releases}*\text{Total volume of assay }b}{\text{ Volume of enzyme taken}*\text{incubation time}} \end{array}$$

### 2.10. Data Analysis

Data analysis was performed using one-way ANOVA in Microsoft Excel 2010. All tests were conducted in triplicate using biological replicates, and the data are expressed as mean ± standard deviation. Graphs were generated using OriginPro 8 software.

## 3. Results and Discussion

### 3.1. Isolation and Screening of Protease-Producing Bacteria

To identify protease-producing bacteria from the soil of the Dire Tannery waste disposal site, a serial dilution technique was employed on nutrient agar media. Based on the size and morphology of the colonies, a total of 36 distinct bacterial isolates were obtained. Previous studies conducted by researchers have focused on isolating and characterizing bacterial cultures for protease production from tannery waste soil samples and effluent. Masi et al. [14] identified 28 different bacterial colonies from a leather industry effluent in Modjo town, Ethiopia. In a similar vein, Saha et al. [15] investigated the protease activity of bacteria associated with tannery waste sites and identified a total of 40 isolates, with 31 displaying positive results for proteolytic activity on various protein-based media. These findings collectively suggest that tannery waste serves as a promising source of bacteria species capable of producing proteases.

In the primary screening for protease-producing bacteria, the identification of proteolytic activity was established by observing the development of a distinct hydrolysis zone surrounding bacterial colonies. Among the 36 isolates examined, a sum of 14 isolates (38.9%) demonstrated protease activity, as evidenced by the formation of clear zones on SMA. Among these, two isolates, namely, DT-5 and DT-15, exhibited larger clear zones measuring 17.00 ± 0.86 mm and 18.00 ± 0.28 mm, respectively, on SMA plates (Table.  1). Similar studies conducted by [16] on tannery wastes from leather processing industries in Hazaribag, Bangladesh, reported skim milk hydrolysis zones ranging from 10.75 to 19.28 mm. In contrast, Singh et al. [3] found that two *Bacillus* bacterial strains, BP1 and BP2, produced narrower hydrolysis zones of 3.5 and 2.2 mm, respectively, from a soil sample. These findings indicate that tannery waste harbors a diverse population of protease-producing bacteria. Since tannery waste is rich in protein yet not well-studied, these bacteria may have developed unique adaptations to survive and efficiently produce proteases. This makes them promising candidates for industrial applications.

Among the isolates, DT-15 exhibited the highest protease activity, providing new insights into *Bacillus* species' enzyme production in this environment. These results could contribute to the development of eco-friendly and cost-effective proteases for industries such as leather processing, detergent production, and food manufacturing.

### 3.2. Secondary Screening of Potential Protease-Producing Bacterial Isolates

**Figure 1:**
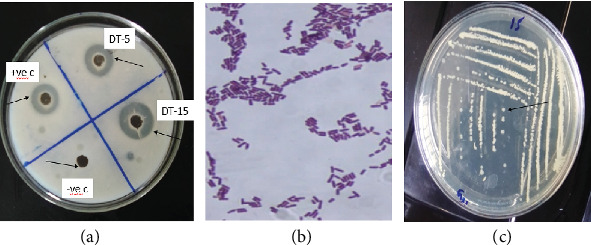
(a) Secondary screening result of the isolates namely (DT-5, DT-15, and C = control) which showed higher clear zone on primary screening (+ve c = positive control *Bacillus cereus* and −ve c = negative control without any isolate). (b) Gram staining viewed under 100x magnification. (c) Bacterial colony (DT-15) using the streak plate method on nutrient agar media after 24 h of incubation at 30°C.

In the secondary screening, two distinct isolates that exhibited larger clear zones during the primary screening were selected for further evaluation. Based on the obtained result DT-15 and DT-5 displayed higher clear zones of 19.00 ± 0.75 mm and 17.5 ± 0.43 mm (Figure 1), respectively, on SMA plates. The formation of the largest clear zone indicated the highest efficiency of this isolate in degrading casein. Therefore, based on the formation of larger clear zone, isolated DT-15 was selected for further protease production studies. A similar study conducted by Verma and Baiswar [17] identified 23 bacteria capable of producing protease on milk agar from treated tannery effluent. Among these isolates, nine exhibited clear zones greater than 20 mm. The present study adds new insights into protease production from bacteria isolated specifically from an untreated tannery waste disposal site, an environment with high protein content but limited exploration. These findings suggest that tannery waste could serve as a valuable reservoir for industrially relevant protease-producing bacteria, with DT-15 emerging as a promising candidate for further optimization and application in biotechnological processes.

### 3.3. Morphological and Molecular Characterization of the Best Isolate

To identify the bacteria, different morphological characterizations were conducted. The colony morphology of the isolate DT-15, including size, color, elevation, and surface margins, was observed on agar plates (Table 2). As shown in Figure 1 the isolate DT-15 exhibited a small colony size, white color, circular shape, rough surface appearance, and flat colony elevation. Cellular morphology observed using Gram staining showed that the isolate had rod shapes with Gram-positive characteristics.

Based on the molecular identification, sequence alignment of isolate DT-15 made with existing 16S rRNA sequence of other bacterial strains in GenBank using the BLAST program, the selected isolate DT-15 was found to be *Bacillus pumilus.* A sequence length of 1193 bp was submitted to GenBank (http://www.ncbi.nlm.nih.gov/) and assigned the accession number PP488878. 16SrRNA gene sequences similarity indicates, *B. pumilus* DT-15 exhibited the highest similarity (97.01%) with the 16S rRNA gene *Bacillus zhangzhouensis* strain Y135. Based on phenotypic and genotypic characteristics, Liu et al. [18] reported two novel strains of *B. zhangzhouensis* sp. Nov *and Bacillus australimaris* sp. Nov. Phylogenetic analyses based on 16S rRNA gene sequences indicated that the two strains belonged to the *B. pumilus* clade. *B. pumilus* is a large group of *Bacillus* that include *B. pumilus*, *Bacillus safensis*, *Bacillus altitudinis, Bacillus xiamenensis*, *B. zhangzhouensis*, and *B. australimaris*. Among *B. pumilus* group 66% discovered in marine sediments, 26% in marine water, and 8% from marine animals [19]. However, the current study indicates that the present isolate could be a new nonmarine source of *B. pumilus*.

**Figure 2:**
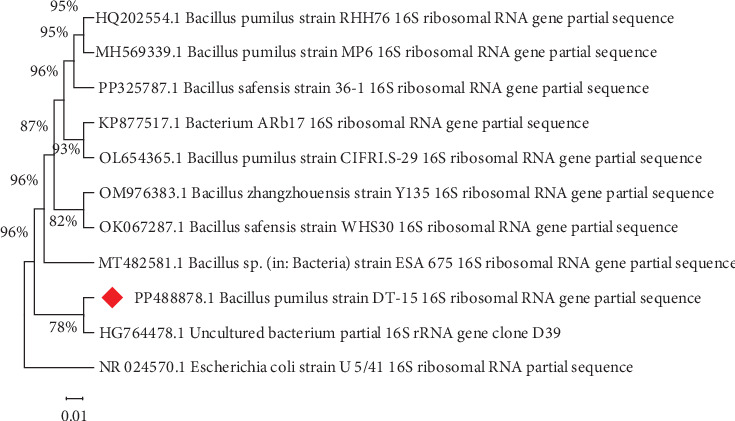
The phylogenetic tree was constructed based on the 16S rRNA gene sequences of *Bacillus pumilu*s strain DT-15. The evolutionary history was inferred using the Maximum Likelihood method and the Tamura 3-parameter model [23]. The percentage of trees in which the associated taxa clustered together is shown next to the branches. Initial tree(s) for the heuristic search were automatically obtained by applying the neighbor-joining (NJ) and BioNJ algorithms to a matrix of pairwise distances estimated using the maximum composite likelihood (MCL) approach. The topology with the highest log-likelihood value was then selected. This analysis involved 11 nucleotide sequences, with codon positions included as 1st, 2nd, 3rd, and noncoding. The final dataset comprised 1520 positions. Evolutionary analyses were conducted using MEGA11 [24]. *Escherichia coli* strain U5/41 was employed as an out-group in the construction of the tree.

Members of the *Bacillus* genus are known for their diverse enzymatic repertoire, with protease being of notable industrial significance. Consistent with this observation, various researchers have documented protease production by distinct strains of *B. pumilus*. Sangeetha and A. G. and I. A. [20] reported *B. pumilus* SG2, isolated from food industry effluent, as a promising source of protease enzyme. Likewise, Rahman et al. [21] identified a novel organic solvent-tolerant protease in *B. pumilus* strain 115b, highlighting it as a top protease producer. Furthermore, Özçelik et al. [22] conducted 16S rRNA sequencing and phylogenetic tree analysis, identifying protease-producing bacteria, particularly *B. pumilus* strain D3. To visualize the genetic relationships, a phylogenetic tree was generated, incorporating the 16S rRNA gene sequences of isolate DT-15 (identified as *B. pumilus*), along with other pertinent nucleotide sequences (Figure 2).

### 3.4. Optimization for Protease Production Media

Optimization of protease production media was carried out for isolates of DT-15 (*B. pumilus* DT-15) using one variable at a time method.

#### 3.4.1. Effect of pH on Protease Production

**Figure 3:**
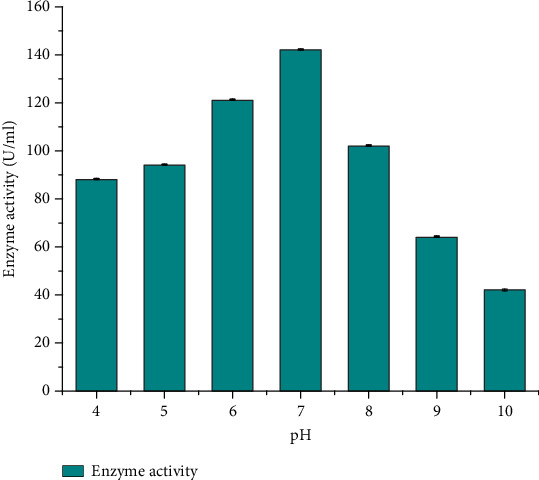
The effect of different pH on protease production of *Bacillus pumilus* DT-15.

As shown in Figure 3, the effect of pH on protease production by isolate DT-15 was investigated across a pH spectrum ranging from 4 to 10. The optimum pH for DT-15 was identified as pH 7, with an enzyme activity of 142.5 ± 0.076 U/mL, indicating a preference for neutral pH conditions. Protease production increased slightly until reaching the optimum pH, after which it declined. Similar patterns have been observed in studies on neutral protease production by *B. subtilis* [25], *B. subtilis* UBT7 [26], and *Bacillus* SNR01 [5]. In the present study, the minimum protease activity was recorded at pH 10, with a value of 42 ± 0.35 U/mL. Comparable findings have been reported by other researchers regarding neutral protease production by different *Bacillus* species. For instance, Pant et al. [27] observed the optimum protease activity of *B. subtilis* at pH 7.4, with an enzyme activity of 143.73 U/mL. Similarly, El-Safey and Abdul-Raouf [28]) found the optimum pH for *B. subtilis* to be pH 7, with an enzyme activity of 177.83 U/mL. In contrast, Sangeetha and A. G. and I. A. [20] reported the optimum pH for the protease-producing *B. pumilus* SG2 as 8.0, and Wang et al. [29] also found an optimum pH of 9.0 for *B. pumilus* BA06. Overall, the optimum pH for protease production by isolate DT-15 (*B. pumilus*) was determined to be pH 7, highlighting its preference for neutral pH conditions.

#### 3.4.2. Effect of Temperature on Protease Activity

**Figure 4:**
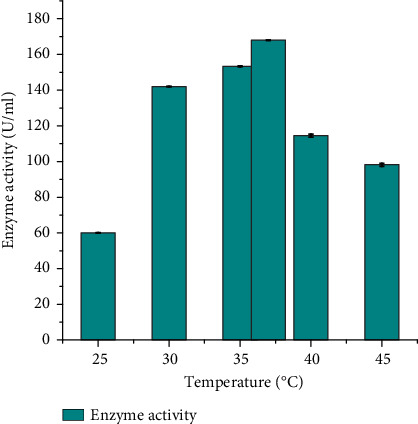
The effect of different incubation temperatures on protease production of *Bacillus pumilus* DT-15.

The protease activity of the DT-15 isolate (identified as *B. pumilus*) was evaluated across a range of temperatures (25°C–45°C) and the result presented in Figure 4. The optimum protease production was recorded at 37°C, with a value of 168 ± 0.02 U/mL. The isolate exhibited efficient protease production within the temperature range of 30°C–37°C. This finding aligns with the results reported by [25], who identified the optimum temperature for protease production by *B. subtilis* as 37°C. Correspondingly, Lakshmi et al. [30] and Das and Prasad [31] documented that 37°C represented the optimum temperature for protease production by *B. subtilis* and *Bacillus licheniformis*, respectively. However, different investigators have reported varying optimal temperatures for protease production from *B. pumilus*. For instance, Wang et al. [29] determined 50°C to be the optimum temperature for protease produced by *B. pumilus* BA06. Moreover, Pan et al. [32] identified 28°C optimum temperature for protease production from *B. pumilus* LYMC-3.Various investigations have delved into the correlation between temperature and protease production, revealing a diverse range of optimal temperatures (25°C–70°C or more) contingent on factors such as the organism, growth conditions, and enzyme type.

#### 3.4.3. Effect of Carbon Sources on Protease Production

**Figure 5:**
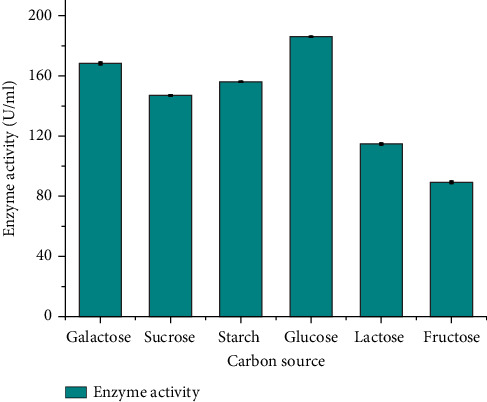
Effect of different carbon sources on protease production of *Bacillus pumilus* DT-15.

As shown in Figure 5, the effect of various carbon sources on protease production was investigated, and the findings revealed that the maximum protease production (186 ± 0.043 U/mL) was attained when glucose used as the carbon source. The data also revealed that all carbon sources tested had a stimulating effect on protease production, with enzyme activity ranging from 89.3 ± 0.57 to 186 ± 0.043 U/mL. These results align with the findings from a study conducted by Hamza and Woldesenbet [33] on protease production by *Bacillus* sp. CAMA14. In their investigation, various carbon sources, including glucose, starch, lactose, maltose, sucrose, and mannitol, were employed. The study noted a substantial improvement in protease production with the addition of glucose.

In contrast, Das and Prasad [31] explored the impact of carbon sources on protease production by *B. subtilis* and found that dextrose was deemed a more favorable carbon source in comparison to glucose. It is important to note that diverse bacterial strains may showcase variations in their preferences for carbon sources in the context of protease production. Furthermore, additional researchers have documented lactose as the favored carbon source for protease production by *B. pumilus* D3 [22]. These differences in carbon source preferences can be attributed to variations in bacterial strains, metabolic pathways, and enzyme regulation mechanisms. Overall, the present study revealed that glucose emerged as the most efficient carbon source for protease production by isolate DT-15 (*B. pumilus*). Nonetheless, it is important to acknowledge that the selection of a carbon source might vary based on the particular bacterial strain and experimental conditions.

#### 3.4.4. Effect of Nitrogen Sources on Protease Production

**Figure 6:**
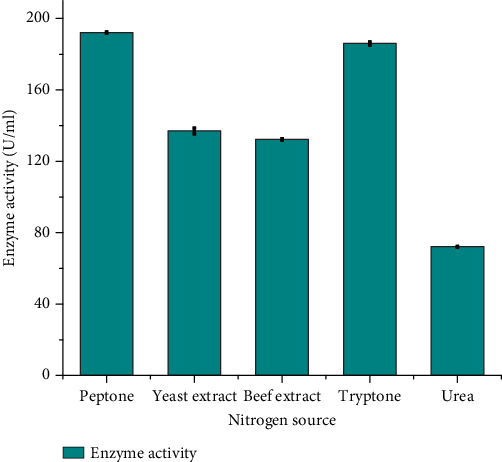
Effect of different nitrogen sources on protease production of *Bacillus pumilus DT-*15.

The production of protease is significantly influenced by nitrogen sources, as nitrogen is metabolized to generate crucial components such as amino acids, nucleic acids, and proteins, all of which are essential for the synthesis of enzymes. Numerous studies in the literature confirm that various nitrogen sources play a role in regulating enzyme production. In the specific case of *B. pumilus* DT-15, peptone emerged as the most effective nitrogen source for protease production, yielding an enzyme activity of 192.2 ± 0.4 U/mL. Following closely, tryptone ranked as the second most influential nitrogen source, exhibiting an enzyme activity of 186 ± 0.57 U/mL. Conversely, the utilization of urea as a nitrogen source resulted in the lowest protease activity, with a recorded value of 72.8 ± 0.28 U/mL (Figure 6).

The findings of this study underscore the promoting impact of peptone as a nitrogen source on protease production, resulting in optimal enzyme performance. The diminished levels of protease production associated with particular nitrogen sources could be ascribed to the bacterial isolates' limited ability to efficiently utilize these nitrogen sources or the inhibitory effects exerted by specific nitrogen sources.

The same findings were also reported by Kumar [34] and Das and Prasad [31] who reported that peptone was a better nitrogen sources for protease production by *B. subtilis.* On the other hand, Nagar et al. [35] reported that beef extract was an optimum nitrogen source for *B. pumilus* SV-85S. Sangeetha and A. G. and I. A. [20] also found that casein and gelatin exhibited a prominent effect on the yield of protease by *B. pumilus* SG 2. Overall, the findings emphasize the importance of selecting suitable nitrogen sources to augment protease production. For *B. pumilus* DT-15, peptone and tryptone emerged as favorable nitrogen sources, fostering elevated levels of protease activity in comparison to urea.

#### 3.4.5. Effect of Incubation Period on Protease Production

**Figure 7:**
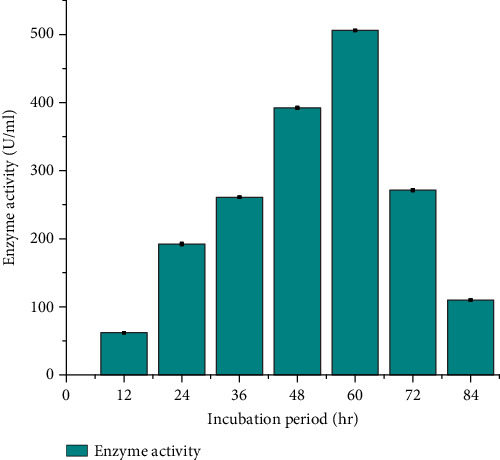
Effect of incubation time from 12 to 84 h on protease production of *Bacillus pumilus* DT-15.

As shown in Figure 7, the influence of varying incubation periods on protease production was explored, revealing that *B. pumilus* DT-15 reached its optimum protease production (506 ± 0.037 U/mL) after 60 h of incubation. Conversely, the minimum protease production (62 ± 0.014 U/mL) was observed at an incubation period of 12 h. These findings are consistent with similar studies conducted by Johnvesly et al. [36] which identified an optimal incubation time of 60 h for two *Bacillus* species, *Bacillus anthraceis* strain S-44 and *Bacillus cereus* strain S-98. In that study, the reported protease activities were 126.09 U/mL and 240.45 U/mL, respectively, further supporting the consistency of the observed incubation period's impact on protease production. Other researchers have reported different optimum incubation times for protease production by different strain of *B. pumilus*. For instance, Sangeetha and A. G. and I. A. [20] and Nagar et al. [35] found the optimum incubation time to be 36 h for *B. pumilus* SG 2 and *B. pumilus* SV-85S. Additionally, Wang et al. [29] reported a maximum protease production at about 36–48 h of culture growth by *B. pumilus* strain BA06. Generally, the results illustrate a general trend where enzyme activity tends to increase with prolonged incubation times until reaching an optimum, beyond which a slight decline is noted. The data further suggest that incubation periods below 60 h decrease the likelihood of successful protease production, while protease production rates slow down as the incubation period exceeds 84 h, possibly due to a loss of catalytic activity over time. Therefore, selecting an appropriate incubation time is crucial for achieving maximum enzyme production. For *B. pumilus* DT-15, the study determined that an incubation period of 60 h proved to be optimal for achieving optimum protease production.

## 4. Conclusions

In this study, we successfully isolated and identified a new nonmarine *B. pumilus* DT-15, a protease-producing bacterium from a tannery waste disposal site. Among the bacterial isolates capable of producing protease, DT-15 was selected as the most effective strain based on its high protease activity. Optimization experiments revealed that DT-15 exhibited maximum protease production after 60 h of incubation at 37°C and pH 7, with peptone and glucose being the best nitrogen and carbon sources, respectively. Notably, the crude protease displayed high specific enzyme activity, suggesting its strong potential for industrial applications. This study highlights the unique adaptability of *B. pumilus* DT-15 to tannery waste conditions, demonstrating its ability to produce high-yield protease in an extreme environment. The findings emphasize that tannery waste disposal sites can serve as valuable reservoirs for industrially significant microorganisms. Furthermore, this is the first study to optimize protease production from *B. pumilus* isolated from a tannery waste site, offering a cost-effective and sustainable source of industrial protease. The versatility of this enzyme makes it a promising candidate for applications in leather processing, detergent formulation, and food production. Future research should focus on scaling up production, enhancing enzyme stability, and evaluating its performance in industrial settings to maximize its biotechnological potential.

## Figures and Tables

**Table 1:** Zone of casein hydrolysis obtained from primary screening result on skim milk agar after a 24-h incubation period.

**S. no.**	**Isolate code**	**Zone of hydrolysis**
1	DT-5	17.00 ± 0.86 mm
2	DT-7	7 ± 0.57 mm
3	DT-9	6.00 ± 0.28 mm
4	DT-14	8.00 ± 0.76 mm
5	DT-15	18.00 ± 0.28 mm
6	DT-17	12.00 ± 1.15 mm
7	DT-20	11.5 ± 1.32 mm
8	DT-22	12.00 ± 0.76 mm
9	DT-25	14.00 ± 1.04 mm
10	DT-27	4.5 ± 0.5 mm
11	DT-29	5.00 ± 0.0.57 mm
12	DT-30	7.00 ± 1.52 mm
13	DT-31	6.00 ± 0.5 mm
14	DT-35	5.00 ± 1.6 mm

Values are the means of triplicate determinations ± standard deviation.

**Table 2:** Morphological characteristics of isolate DT-15.

**Colony morphology**	**Isolate DT-15**
Gram staining	+
Motility test	+
Endospore test	+
Size	Small
Color	White
Elevation	Flat
Shape	Circular
Surface	Rough

Key: + indicates positive result for a given morphological identification.

## Data Availability

The data that support the finding of this study are available from the corresponding author upon reasonable request.

## References

[1] Palsaniya P., Mishra R., Beejawat N., Sethi S., Gupta B. L. (2012). Optimization of Alkaline Protease Production From Bacteria Isolated From Soil. *Journal of Microbiology and Biotechnology Research*.

[2] Abrar T. (2017). Bacterial Protease Enzyme: Safe and Good Alternative for Industrial and Commercial Use. *International Journal of Chemical and Biomolecular Science*.

[3] Singh P., Rani A., Chaudhary N. (2015). Isolation and Characterization of Protease Producing *Bacillus sp* From Soil. *International Journal of Pharma Sciences and Research (IJPSR)*.

[4] Sharma K. M., Kumar R., Vats S., Gupta A. (2014). Production, Partial Purification and Characterization of Alkaline Protease From *Bacillus aryabhattai* K3. *International Journal of Advances in Pharmacy, Biology and Chemistry*.

[5] Josephine F. S., Ramya V. S., Devi N. (2012). Isolation, Production and Characterization of Protease From Bacillus sp. Isolated From Soil Sample. *Journal of Microbiology and Biotechnology Research*.

[6] Kasana R. C., Salwan R., Yadav S. K. (2011). Microbial Proteases : Detection, Production, and Genetic Improvement. *Critical Reviews in Microbiology*.

[7] Patel Y., Gupte A., Gupte S. (2018). Production, Partial Purification, Characterization and Detergent Compatibility of Alkaline Protease from Soil Isolate *Bacillus cereus* AG1. *International Journal of Current Microbiology and Applied Sciences*.

[8] Coico R. (2006). Gram Staining. *Current Protocols in Microbiology*.

[9] Shields P., Cathcart L. (2011). Motility Test Medium Protocol. *American Society for Microbiology*.

[10] Oktari A., Supriatin Y., Kamal M., Syafrullah H. (2017). The Bacterial Endospore Stain on Schaeffer Fulton using Variation of Methylene Blue Solution. *Journal of Physics: Journal of Physics: Conference Series*.

[11] Wright M. H., Adelskov J., Greene A. C. (2017). Bacterial DNA Extraction Using Individual Enzymes and Phenol/Chloroform Separation. *Journal of Microbiology & Biology Education*.

[12] Lowry O. H., Rosebrough N. J., Farr A. L., Randall R. J. (1951). Protein Measurement With the Folin Phenol Reagent. *Journal of Biological Chemistry*.

[13] Orhan E., Omay D., Güveniur Y. (2005). Partial Purification and Characterization of Protease Enzyme From *Bacillus subtilis* and *Bacillus cereus*. *Twenty-Sixth Symposium on Biotechnology for Fuels and Chemicals*.

[14] Masi C., Gemechu G., Tafesse M. (2021). Isolation, Screening, Characterization, and Identification of Alkaline Protease-Producing Bacteria From Leather Industry Effluent. *Annals of Microbiology*.

[15] Saha M. L., Begum K. J. M. H., Khan M. R., Gomes D. J. (2012). Bacteria Associated With the Tannery Effluent and Their Alkaline Protease Activities. *Plant Tissue Culture and Biotechnology*.

[16] Rahman M. S., Islam M. R., Mondol O. K., Rahman M. S., Sabrin F., Zohora U. S. (2018). Screening of Protease Producing Bacteria From Tannery Wastes of Leather Processing Industries at Hazaribag, Bangladesh. *Jahangirnagar University Journal of Biological Sciences*.

[17] Verma T., Baiswar V. (2013). Isolation and Characterization of Extracellular Thermoalkaline Protease Producing *Bacillus cereus* Isolated From Tannery Effluent. *International Journal Environmental Science*.

[18] Liu Y., Lai Q., Du J., Shao Z. (2016). Bacillus zhangzhouensis sp. nov. and Bacillus australimaris sp. nov. *International Journal of Systematic and Evolutionary Microbiology*.

[19] Fu X., Gong L., Liu Y., Lai Q., Li G., Shao Z. (2021). *Bacillus pumilus* Group Comparative Genomics: Toward Pangenome Features, Diversity, and Marine Environmental Adaptation. *Frontiers in Microbiology*.

[20] Sangeetha R., Geetha A., Arulpandi I. (2008). Optimization of Protease and Lipase Production by Bacillus Pumilus SG 2 Isolated from an Industrial Effluent. *Internet Journal of Microbiology*.

[21] Rahman R. N. Z. R. A., Mahamad S., Salleh A. B., Basri M. (2007). A New Organic Solvent Tolerant Protease From *Bacillus pumilus* 115b. *Journal of Industrial Microbiology and Biotechnology*.

[22] Özçelik B., Aytar P., Gedikli S., Yardimci E., Çalişkan F., Çabuk A. (2014). Production of an Alkaline Protease Using *Bacillus pumilus* D3 Without Inactivation by SDS, Its Characterization, and Purification. *Journal of Enzyme Inhibition and Medicinal Chemistry*.

[23] Tamura K., Stecher G., Kumar S. (2021). MEGA 11: Molecular Evolutionary Genetics Analysis Version 11. *Molecular Biology and Evolution*.

[24] Tamura K. (1992). Estimation of the Number of Nucleotide Substitutions When There Are Strong Transition-Transversion and G + C-Content Biases. *Molecular Biology and Evolution*.

[25] Padmapriya M., Williams B. C. (2017). Purification and Characterization of Neutral Protease Enzyme From *Bacillus subtilis*. *Journal of Microbiology and Biotechnology Research*.

[26] Prihanto A. A., Jaziri A. A., Perwira I. Y. (2016). Purification and Characterization of Neutral Protease From *Bacillus substilis* UBT7 Isolated From Terasi, Indonesian Fermented Fish. *Biosciences Biotechnology Research Asia*.

[27] Pant G., Prakash A., Pavani J. V. P. (2015). Production, Optimization and Partial Purification of Protease *From Bacillus subtilis*. *Integrative Medicine Research*.

[28] El-Safey E. M., Abdul-Raouf U. M. (2004). Production, Purification, and Characterization of Protease Enzyme From *Bacillus subtilis*. *International Conferences for Development and the Environment in the Arab World*.

[29] Wang H. Y., Liu D. M., Liu Y. (2007). Screening and Mutagenesis of a Novel Bacillus pumilus Strain Producing Alkaline Protease for Dehairing. *Letters in Applied Microbiology*.

[30] Lakshmi B. K. M., Sri P. V. R., Devi K. A., Hemalatha K. P. J. (2014). Media Optimization of Protease Production by *Bacillus licheniformis* and Partial Characterization of Alkaline Protease. *International Journal of Current Microbiology and Applied Sciences*.

[31] Das G., Prasad M. P. (2015). Isolation, Purification & Mass Production of Protease Enzyme From *Bacillus subtilis*. *International Research Journals of Microbiology*.

[32] Pan M., Wang Y., Tan J., Liu F., Hu J. (2023). Optimization of Fermentation Conditions for *Bacillus pumilus* LYMC-3 to Antagonize *Sphaeropsis sapinea*. *Fermentation*.

[33] Hamza T. A., Woldesenbet F. (2017). Optimization of Culture Growth Parameters for Production of Protease From Bacteria, Isolated From Soil. *Bioscience and Bioengineering*.

[34] Kumar C. G. (2002). Purification and Characterization of a Thermostable Alkaline Protease From Alkalophilic Bacillus pumilus. *Letters in Applied Microbiology*.

[35] Nagar S., Gupta V. K., Kumar D., Lalit Kumar R. C. K. (2010). Production and Optimization of Cellulase-Free, Alkali-Stable Xylanase by *Bacillus pumilus* SV-85S in Submerged Fermentation. *Journal of Industrial Microbiology & Biotechnology*.

[36] Johnvesly B., Manjunath B. R., Naik G. R. (2002). Pigeon Pea Waste as a Novel, Inexpensive, Substrate for Production of a Thermostable Alkaline Protease From Thermoalkalophilic Bacillus sp. JB-99. *Bioresource Technology*.

